# Retraction Note: Relationship between fear of evaluation, ambivalence over emotional expression, and self-compassion among university students

**DOI:** 10.1186/s40359-025-03903-6

**Published:** 2026-01-05

**Authors:** Tian Huang, Wenbo Wang

**Affiliations:** School of Physical Education and Sports, Sichuan University, Chengdu, Sichuan Province China

**Retraction Note: BMC Psychology 12, 128 (2024)**

**https://doi.org/10.1186/s40359-024-01629-5**

The Editor has retracted this article after post-publication review found several major issues, including but not limited to the following:

• Self-compassion and self-care are used interchangeably in the abstract despite being two distinct terms-;-.

• The number of the items that are commonly used to measure self-compassion are typically 26 and not 25. In the study, one item was excluded without providing a reason for the exclusion, which may affect the interpretation of the results;

• There appear to be methodological issues and inconsistencies between the text of the article and the data presented.

The authors did not respond to a request to clarify those issues. The Editor therefore has lost confidence in the integrity of the results presented in the article.

The authors did not respond to correspondence from the Editor about this retraction.

